# Biopsychosocial factors of quality of life in individuals with moderate to severe traumatic brain injury: a scoping review

**DOI:** 10.1007/s11136-023-03511-0

**Published:** 2023-11-05

**Authors:** Rinni Mamman, Jasleen Grewal, Juliana Nicole Garrone, Julia Schmidt

**Affiliations:** 1 Graduate Program in Rehabilitation Sciences, Faculty of Medicine, University of British Columbia, Vancouver, Canada; 2 Rehabilitation Research Program, Centre for Aging SMART, Vancouver Coastal Health Research Institute, Vancouver, Canada; 3 Fraser Health Authority, Vancouver, Canada; 4 Department of Occupational Science and Occupational Therapy, Faculty of Medicine, University of British Columbia, Vancouver, BC Canada

**Keywords:** Traumatic brain injury, Quality of life, Biopsychosocial model, Scoping review

## Abstract

**Purpose:**

Individuals with moderate to severe traumatic brain injury (TBI) experience changes in their quality-of-life (QOL) post-injury. Given the vast literature that exists about QOL after TBI, a scoping review was performed to identify the different biopsychosocial factors that affect a person’s QOL after a moderate to severe TBI.

**Methods:**

A scoping review was conducted using the following electronic databases: MEDLINE, CINAHL, Embase, and PsycINFO. Terms relating to TBI and QOL were used.

**Results:**

There were 7576 articles obtained from the databases, resulting in 535 full-text articles. Ultimately, 52 articles were extracted, which consisted of biopsychosocial QOL factors after TBI. The biopsychosocial factors of QOL after TBI included 19 biological factors (i.e., sex, TBI severity, cognition), 16 psychological factors (i.e., depression, self-efficacy, coping styles), and 19 social factors (i.e., employment, social participation, social support). Factors such as fatigue, self-awareness, transition, and discharge from hospitals are known issues in TBI literature but were minimally reported in studies in this review, identifying them as potential gaps in research.

**Conclusion:**

Identifying biopsychosocial factors relating to QOL after TBI can enable health services to develop targeted rehabilitation programs for individuals with TBI.

**Supplementary Information:**

The online version contains supplementary material available at 10.1007/s11136-023-03511-0.

## Plain English summary

After traumatic brain injury, people can experience changes in quality-of-life. Quality-of-life may be defined as a person’s overall perception of their life, with regard to their health, expectations, and external influences. Although a lot of research has been conducted on this topic, a summary of this research is needed to provide information to clinicians, researchers, and individuals with brain injuries, to help improve quality-of-life after a traumatic brain injury. In this study, we conducted an extensive search of past literature and identified the different biological, psychological, and social factors of quality-of-life after a traumatic brain injury. The results describe the influence of factors, such as mental health and social support, on quality-of-life after traumatic brain injury. Findings can guide health services to tailor their rehabilitation treatments to help improve the lives of individuals with traumatic brain injury.

## Background

Traumatic brain injury (TBI) is sustained by approximately 69 million individuals each year worldwide [1]. Individuals with TBI can face long-term problems that impact their health, function, and daily life [2]. These may arise from the injury itself (i.e., mobility and cognitive issues), as well as from barriers in their surroundings (i.e., environmental and societal) [3, 4]. These problems can have a major impact on an individual’s quality-of-life (QOL) post-injury. QOL is an important outcome, with increasing interest in the investigation and implementation of strategies to improve QOL after TBI.

QOL is a broad construct with varying definitions. The World Health Organization describes QOL as having an individualized component, with subjective experiences shaping a person’s perception of life within their cultural context, and in relation to their personal goals, expectations, and interests [5]. Given this expansive definition, QOL can be described through the physical health, psychological, social relationships, and environmental domains, and are central in providing insight into an individual’s QOL [6]. These domains align with the biological, psychological, and social changes that are typically experienced by individuals with TBI. As QOL encompasses a broad range of aspects of human life, a more comprehensive measure known as the health-related QOL (HRQOL) was introduced. HRQOL is a multi-dimensional construct that measures how an individual perceives the effects of their injury on their physical, mental, and social function [7]. HRQOL is often used as an outcome measure for individuals with chronic illnesses to signify their QOL.

TBI can be defined as a chronic condition, as the problems that stem from a TBI can occur at any point post-injury [8]. There may be a low rate of full recovery after a moderate to severe TBI as the effects are often long lasting, making the navigation of life after TBI an ongoing process and impacting a person’s QOL [9]. As these changes post-TBI can affect a person in multiple ways, a biopsychosocial perspective can facilitate the understanding on how to improve QOL, which is a primary goal in TBI rehabilitation. Therefore, using a biopsychosocial framework enables the development and implementation of interventions needed to manage the challenges experienced after a TBI.

There have been a few reviews to date identifying factors exploring QOL and TBI [10]. One systematic review reported on the nature and predictors of QOL that affected children and adolescents with TBI [11]. Another review described the differences in QOL between individuals with and without a TBI, with studies reporting individuals with TBI having a lower QOL [12]. Finally, a systematic review identified 16 studies that assessed at least two of the four QOL domains (physical, social, environmental, cognitive) experienced post-injury [13]. However, despite the growing evidence of the numerous factors affecting QOL after a moderate to severe TBI, no recent reviews have been conducted.

Past reviews focus too broadly on QOL and have failed to explore in detail the biological, psychological, and social factors in the adult TBI population. As such, despite the growing number of research studies about these different factors in the TBI population, there have been no recent reviews conducted to provide a better understanding of the factors associated with QOL from a biopsychosocial perspective. Thus, the primary objective of this scoping review is to identify QOL factors and analyze potential knowledge gaps in QOL research in the moderate to severe adult TBI population with relation to biological, psychological, and social domains.

## Methods

A scoping review was conducted, in accordance with the framework by Arksey and O’Malley [14] and Levac et al. [15]. Five stages were included in this scoping review: identifying the research question, identifying relevant studies, choosing studies, charting the data, and reporting the results [14]. This scoping review is reported using the PRISMA Extension for Scoping Reviews [16].

### Search strategy

The articles were identified from the following databases: MEDLINE, CINAHL, Embase, and PsycINFO, with the search conducted on 24 April 2021. With the help of a subject-area expert librarian, the initial database search was developed using key search terms: ‘brain injuries,’ ‘quality of life,’ and ‘life quality.’ Boolean terms (‘AND,’ ‘OR,’ ‘NOT’) were used to combine terms, and asterisks were used to include variants in spelling.

### Eligibility criteria

English peer-reviewed articles published since 1990 to the date of search were reviewed to limit the breadth of articles obtained and to analyze more recent QOL literature. Full-text articles were selected if they had: (1) presented data on half or more participants with a moderate to severe TBI who were 18 years or older, (2) identified biological, psychological, or social QOL factors, and (3) provided quantitative data on a validated global QOL measure. Adolescents or children with TBI were not included as there may be developmental causes affecting their QOL, which may not be present in the adult population. Furthermore, the exclusion criteria consisted of articles with no full-texts available (i.e., conference abstracts, poster, dissertations) or study designs such as case studies or qualitative methodologies. Articles with surgical (e.g., cranioplasty) or hormone-related interventions (e.g., pituitary dysfunction) were also excluded.

### Data screening and extraction

The study selection and information extraction were performed using the software Covidence [17]. All authors were involved in each stage of the review process, with additional help of four research assistants. Authors RM, JG, JNG, and two research assistants independently performed the title and abstract screening, while authors RM, JNG, and JS independently conducted the full-text screening. Conflicts were resolved by JS. Data extraction was independently conducted by RM, JG, and two research assistants. In line with the aims of this scoping review, a critical appraisal was not conducted. Extracted data included the country of the study, authors, participant information, measures of QOL, factors associated with QOL, and other main findings (Tables 1, 2).

**Table 1:** Demographic information of included studies

Reference and country	Design	Number of participants	Rehabilitation setting	Age group	QOL outcome measure
Alway et al., (2016) Australia	Prospective longitudinal	*N* = 203 TBI = 203 Males = 159 Females = 44	Inpatient	Mean age = 34.4 years	QOLI
Andelic et al., (2009) Norway	Retrospective longitudinal	*N* = 62 TBI = 62 Males = 47 Females = 15	Outpatient	Mean age = 40.8 years	SF-36
Andelic et al., (2015) Norway	Prospective longitudinal	*N* = 97 TBI = 97 Males = 76 Females = 21	Outpatient	Mean age at injury = 30.9 years	SF-36
Andelic et al., (2018) Norway	Prospective longitudinal	*N* = 44 TBI = 44 Males = 33 Females = 11	Outpatient	Mean age = 50.8 years	SF-36
Azouvi et al., (2016) France	Prospective longitudinal	*N* = 85 TBI = 85 Males = 69 Females = 16	Outpatient	Mean age = 31.7 years	QOLIBRI
Bosma et al., (2018) Switzerland	Prospective longitudinal	*N* = 108 TBI = 108 Males = 81 Females = 25	Inpatient	Mean age (under 50 years group) = 28.3 years, mean age (older than 50 years group) = 65.92 years	SF-12
Cantor et al., (2008) United States	Cross-sectional	*N* = 308 (including 64 mild TBI and 85 controls) TBI = 105 Males = 151 Females = 157	Outpatient	Mean age (TBI) = 47.8 years	SF-36 and Life-3
Delft-Schreurs et al., (2014) Netherlands	Cross-sectional	*N* = 173 TBI = 173 Males = 120 Females = 53	Outpatient	Mean age = 47 years	WHOQOL-BREF
Diaz et al., (2012) Brazil	Prospective longitudinal	*N* = 33 TBI = 33 Males = 29 Females = 4	Inpatient	Mean age = 31.36 years	SF-36
Douglas (2020) Australia	Cross-sectional	*N* = 23 TBI = 23 Males = 20 Females = 3	Outpatient	Mean age = 36.96 years	Self-rated QOL scale
Esbjörnsson et al., (2013) Sweden	Cross-sectional	*N* = 18 TBI = 18 Males = 9 Females = 9	Not specified	Age range = 19 to 62 years	EQ-5D and EQ-VAS
Farmer et al., (2003) United States	Cross-sectional	*N* = 56 TBI = 56 Males = 29 Females = 27	Outpatient	Mean age = 38 years	QOL scale
Forslund et al. (2013) Norway	Prospective longitudinal	*N* = 91 TBI = 91 Males = 70 Females = 21	Outpatient	Mean age = 31.1 years	SF-36
Forslund et al., (2021) Norway	Prospective longitudinal	*N* = 97 TBI = 97 Males = 76 Females = 21	Outpatient	Mean age = 30.3 years	SF-36
Gaertner et al. (2020) Switzerland	Prospective longitudinal	*N* = 174 TBI = 174 Males = 132 Females = 42	Outpatient	Mean age = 51 years	SF-12
Genova et al. (2017) United States	Case–control	*N* = 74 (including 2 mild TBI, 1 missing severity, 27 controls) TBI = 44 Males = N/S Females = N/S	Not specified	Mean age (TBI) = 39.17 years	Health Status Questionnaire
Gorgoraptis et al., (2019) United Kingdom	Retrospective cross-sectional	*N* = 240 (including 41 mild TBI, 27 symptomatic TBI) TBI = 172 Males = 174 Females = 66	Outpatient	Age range = 22–91 years	SF-36
Gould et al., (2011) Australia	Prospective longitudinal	*N* = 122 TBI = 122 Males = 96 Females = 26	Outpatient	Mean age at injury = 34.89 years	QOLI
Gould et al., (2015) Australia	Prospective longitudinal	*N* = 95 Males = 75 Females = 20 TBI = 95	Outpatient	Mean age at injury = 38.2 years	QOLI
Goverover et al., (2014) United States	Cross-sectional	*N* = 30 (5 mild TBI, 4 undetermined severity) TBI = 21 Males = 20 Females = 10	Outpatient	Mean age = 40.03 years	SF-12
Goverover et al., (2017) United States	Case–control	*N* = 82 (including 30 controls) TBI = 52 Males = 33 Females = 19	Outpatient	Mean age (TBI) = 39.1 years	SF-36
Grauwmeijer et al., (2014) Netherlands	Prospective longitudinal	*N* = 97 TBI = 97 Males = 70 Females = 27	Inpatient and outpatient	Mean age = 32.8 years	SF-36
Grauwmeijer et al., (2018) Netherlands	Prospective longitudinal	*N* = 50 TBI = 50 Males = 34 Females = 16	Inpatient and outpatient	Age range at injury = 16 to 67 years	SF-36
Gregorio et al., (2014) Australia	Prospective longitudinal	*N* = 174 (including 22 mild TBI) TBI = 152 Males = 139 Females = 35	Inpatient and outpatient	Mean age at injury = 34.3	QOLI
Henry et al., (2006) United Kingdom	Case-control	*N* = 59 (including 31 controls) TBI = 28 Males (TBI) = 22 Females (TBI) = 6	Outpatient	Mean age = 40.3 years	LEIPAD
Hibbard et al., (2004) United States	Prospective longitudinal	*N* = 188 (including 53 with loss of conscious below 20 min, 17 not specified) TBI = 118 Males = 100 Females = 88	Outpatient	Mean age = 40.4 years	LLATBI, UIN/ Flanagan Scale of Needs, Life-3
Huebner et al., (2003) United States	Retrospective longitudinal	*N* = 25 (including 3 mild TBI) TBI = 22 Males = 17 Females = 8	Outpatient	Mean age = 43.79 years	QOLR
Jacobsson et al., (2010) Sweden	Case-control	*N* = 67 (including 32 mild TBI) TBI = 35 Males = 51 Females = 16	Outpatient	Mean age = 44 years	SF-36
Johnson & Ditchman (2020) United States	Cross-sectional	*N* = 183 (including 33 of acquired brain injury) TBI = 150 Males = 61 Females = 108 (missing sex information for 14 participants)	Outpatient	Mean age = 49 years	SWLS
Kalpakjian et al., (2004) United States	Cross-sectional	*N* = 50 TBI = 50 Males = 32 Females = 18	Outpatient	Mean age = 38.74 years	QOLI
Koskinen et al., (1998) Finland	Prospective longitudinal	*N* = 15 TBI = 15 Males = 12 Females = 3	Inpatient and Outpatient	Age range = 22 to 49 years	Life satisfaction measure
McLean et al., (2014) Canada	Cross-sectional	*N* = 46 TBI = 46 Males = 31 Females = 15	Outpatient	Mean age = 44.17 years	QOLHQ, AKHS, UCLA-LS
Nalder et al., (2012) Australia	Prospective longitudinal	*N* = 127 TBI = 127 Males = 99 Females = 28	Outpatient	Age range = 18 to 60 years	EQ-5D
O'Neill et al., (1998) United States	Cross-sectional	*N* = 337 (including 70 with loss of consciousness below 20 min) TBI = 267 Males = 197 Females = 140	Outpatient	Age range = 18 to 64 years	Bigelow QOL Questionnaire, Global QOL questionnaire
Pettemeridou et al., (2020) Cyprus	Case–control	*N* = 57 (including 24 controls) TBI = 33 Males = 57	Inpatient	Mean age = 31.92 years	WHOQOL-BREF and QOLIBRI
Rauen et al., (2020) Germany	Cross-sectional	*N* = 135 (including 18 mild TBI, 51 not severity unspecified) TBI = 66 Males = 103 Females = 32	Outpatient	Mean age = 53.1 years	QOLIBRI
Rauen et al., (2021) Germany	Cross-sectional	*N* = 135 (including 18 mild, 51 severity not specified) TBI = 66 Males = 102 Females = 33	Outpatient	Mean age (males/females) = 53.08/53.24 years	QOLIBRI
Reddy et al., (2017) India	Cross-sectional	*N* = 60 (including 26 severity not specified) TBI = 34 Males = 54 Females = 6	Inpatient and outpatient	Mean age = 28.27 years	WHOQOL Assessment-BREF
Sashika et al., (2017) Japan	Cross-sectional	*N* = 31 (including 5 mild TBI) TBI = 26 Males = 17 Females = 14	Outpatient	Age range = 18 to 63 years	SF-36
Sasse et al., (2014) Germany	Cross-sectional	*N* = 141 (including 44 mild TBI, 25 complicated mild) TBI = 61 Males = 100 Females = 41	Inpatient	Age range = 17 to 68 years	QOLIBRI, SF-36
Soberg et al., (2013) Norway	Prospective longitudinal	*N* = 126 TBI = 126 Males = 98 Females = 28	Inpatient	Mean age = 38.9 years	QOLIBRI
Steadman-Pare et al. (2001) United States	Retrospective longitudinal	*N* = 275 TBI = 275 Males = 194 Females = 81	Outpatient	Mean age = 43.3 years	Self-rated QOL measure
Takada et al., (2016) Japan	Cross-sectional	*N* = 29 (including 9 mild TBI) TBI = 20 Males = 16 Females = 13	Outpatient	Mean age = 38.8 years	SF-36
Tomberg et al., (2005) Estonia	Case-control	*N* = 153 (including 68 controls) TBI = 85 Males (TBI) = 69 Females (TBI) = 16	Outpatient	Mean age = 37.7 years	RAND-36
Tomberg et al., (2007) Estonia	Prospective longitudinal	*N* = 31 TBI = 31 Males = 25 Females = 6	Outpatient	Mean age = 43.8 years	RAND-36
Tsaousides et al., (2008) United States	Cross-sectional	*N* = 317 (including 108 mild TBI) TBI = 207 Males = 182 Females = 135	Outpatient	Mean age = 40.79 years	LLATBI, Life -3
Tsaousides et al., (2009) United States	Cross-sectional	*N* = 425 (including 98 with loss of consciousness below 20 min) TBI = 205 Males = 237 Females = 188	Outpatient	Mean age = 34.9 years	Life-3, UIN/ Flanagan Scale of Needs
Tsaousides et al., (2011) United States	Cross-sectional	*N* = 356 (including 134 mild TBI) TBI = 222 Males = 186 Females = 170	Outpatient	Mean age = 44.45 years	Life-3
Ulfarsson et al., (2014) Sweden	Retrospective longitudinal	*N* = 51 TBI = 51 Males = 38 Females = 13	Outpatient	Mean age = 37.9 years	SF-36
Vickery et al. (2005) United States	Cross-sectional	*N* = 19 (including 1 mild TBI, 4 with acquired brain injury) TBI = 14 Males = 13 Females = 6	Inpatient	Mean age = 30.3 years	QOLI
Wielenga-Boiten et al., (2015) Netherlands	Prospective longitudinal	*N* = 85 TBI = 85 Males = 59 Females = 26	Inpatient and outpatient	Mean age = 32.1 years	SIP-68
Williams et al., (2012) Australia	Cross-sectional	*N* = 39 TBI = 39 Males = 29 Females = 10	Inpatient	Mean age = 27.7 years	WHOQOL-BREF, AQOL-2

TBI: Traumatic brain injury; QOL: Quality of life; HRQOL: Health-related quality of life; QOLI: Quality of life inventory; SF-36: Short form 36 health survey; SF-12: Short form 12 health survey; QOLIBRI: Quality of life after brain injury; EQ-5D: European questionnaire-5 dimensions; EQ-VAS: European questionnaire visual analog scale; LLATBI: Living life after traumatic brain injury; UIN: Unmet important needs; QOLR: Quality-of-life rating, SWLS: Satisfaction of life scale; QOLHQ: Quality of life and health questionnaire; AKHS: Abdel-Khalek happiness scale; UCLA-LS: University of California at Los Angeles loneliness scale; WHOQOL-BREF: World health organization quality of life (shorter version), SIP-68: Sickness impact profile-68; AQOL-2: Assessment of quality of life-2

**Table 2:** Biopsychosocial factors of included studies

Reference	Biological factors	Psychological factors	Social factors
Alway et al., (2016)		Posttraumatic stress Participants with posttraumatic stress had lower QOLI scores compared to participants with no posttraumatic stress	
Andelic et al., (2009)	Epilepsy Individuals with epilepsy had lower scores for Vitality and Role- Emotional domains Sex Females reported lower QOL scores than males in the Mental Health domain	Depression Individuals with depression scored worse on all SF-36 subscales	Employment Employed participants showed better scores than unemployed participants in the Physical Functioning, Role-Physical, and Role-Emotional domains
Andelic et al., (2015)	Sex Females reported lower scores on Role–Physical, Bodily Pain (more pain) and General Health domains overtime TBI severity Overtime, higher severity of TBI was associated with lower Physical Functioning but higher General Health (only at the first-year follow-up) and Role-Physical domains		Education Participants with a higher level of education reported higher Physical Functioning overtime Employment Unemployment was associated with lower Physical Functioning and General Health domains. Working in a physical job reported worse General Health overtime
Andelic et al., (2018)	Sex Being a female at 10 years predicted poorer mental health at 20 years	Depression Having depression at 10 years predicted worse mental and physical health at 20 years	Community integration (productivity) Engaging in activities (i.e., work, school) at 10 years predicted better physical and mental health at 20 years
Azouvi et al., (2016)	Age Age had an indirect influence on QOL Cognition Cognition was a direct predictor of QOL	Depression/Anxiety Depression/anxiety were direct predictors of QOL Somatic impairments Somatic impairments such as pain, motor, and balance deficits had an indirect influence on QOL	
Bosma et al., (2018)		Posttraumatic stress Symptoms of posttraumatic stress were negatively associated with mental HRQOL	
Cantor et al., (2008)	Fatigue Fatigue negatively correlated with all aspects of the SF-36 for both controls and individuals with TBI. Fatigue was correlated with overall QOL (Life-3) for individuals with TBI		
Delft-Schreurs et al., (2014)	Age Older age was associated with higher QOL in the environmental domain Length of hospital stay Longer hospitalization was associated with lower QOL in the physical domain Length of ICU treatment Longer ICU treatment was associated with lower QOL in the physical domain Pre-injury comorbidity (physical and mental) Having a pre-injury physical comorbidity was associated with lower QOL in the physical domain. Undergoing pre-injury mental treatment was associated with lower QOL in the physical and psychological domains Thoracic injury Thoracic injuries, when compared to other body regions, was associated with better QOL in the environmental domain		Employment Resuming employment and was associated with high QOL on all domains Living with others Living with other individuals was associated with high QOL on all domains
Diaz et al., (2012)		Major depressive disorder Participants with major depressive disorder showed impairment in all SF-36 domains Personality changes Participants with personality changes reported lower scores in the General Health, Physical, and Social Functioning domains of the SF-36	
Douglas (2020)			Number of friends An association was reported between the number of friends and QOL
Esbjörnsson et al., (2013)	Cognition (Cognitive attention, executive functioning) Participants who reported better cognition, attention, and had fewer problems in planning had higher QOL	Depression Participants who were found to be less depressed had better QOL Motivation Participants who were more motivated had better QOL	Socially isolated Participants who were less socially isolated reported higher QOL
Farmer et al., (2003)			Environmental setting Living in a rural setting was a predictor of high QOL Seeking social support Positive appraisals in seeking social support were a predictor of high QOL while hesitation in seeking social support was correlated with low QOL Productive activities Predictors of high QOL were increased time in productive activities
Forslund et al. (2013)	Age Being older than 31 years of age indicated lower scores for the Role-Physical domain. Additionally, younger age was a predictor of higher physical QOL TBI severity Higher severity of TBI was a predictor of higher physical QOL Functional Independence There were differences in QOL between participants who had more functional independence compared to those who had less independence Trauma Participants with less severe overall trauma had worse Bodily Pain scores	Depression Participants with depression reported lower HRQOL in all SF-36 subscales, when compared with participants without depression. Lower depression was a predictor of higher mental QOL Positive change More positive change was a predictor of higher mental and physical QOL	Community integration More community integration was a predictor of higher physical QOL. There were differences in QOL between participants who had more community integration compared to individuals with less integration Education Being educated for more than 12 years showed better Physical Functioning Employment Being employed at the time of injury indicated better HRQOL in all domains
Forslund et al., (2021)	Posttraumatic amnesia Shorter periods of posttraumatic amnesia were predictive of physical health Sex Being a male predicted higher physical and mental health trajectories over the 10-year follow-up Time since injury Over time, both physical and mental health increased		Employment (pre-injury) Being employed pre-injury predicted higher physical and mental health trajectories over the 10-year follow-up
Gaertner et al. (2020)			Relatives’ interpersonal functioning Relatives’ interpersonal functioning was positively associated with mental HRQOL. Additionally, relatives increasing interpersonal functioning was positively associated with physical HRQOL in participants > 50 years
Genova et al. (2017)	Facial affect recognition Better performance on facial affect recognition tasks was associated with poorer social and emotional QoL		
Gorgoraptis et al., (2019)	Cognition Participants with cognitive impairment reported low scores on the Physical Functioning, Social and Emotional Role functioning, and Mental Health subscales. Cognitive impairment was a predictor for poorer HRQOL on the Social and Emotional Role subscale, independent of depression, sleep disturbance, excessive daytime sleepiness, and severity Excessive daytime sleepiness Participants with excessive daytime sleepiness had low HRQOL on all SF-36 domains except Physical Functioning Sleep disturbance Participants sleep disturbance had low HRQOL on all SF-36 domains	Depression Participants with depression had low HRQOL on all SF-36 domains	
Gould et al., (2011)		Anxiety Depression Psychiatric disorder Having an anxiety, depressive, or a psychiatric disorder, at 12 months post- injury was associated with poorer QOL	
Gould et al., (2015)		Positive changes Participants with positive changes in life post- TBI had higher QOL scores at 6-, 12-, and 24-months post-injury	
Goverover et al., (2014)		Depression Depression was associated with lower HRQOL Self-awareness Higher levels of self-awareness were associated with poorer HRQOL	
Goverover et al., (2017)	Time since injury Time since injury is a predictor of HRQOL	Depression Depression is a predictor of HRQOL	Activities (everyday) More current and retained activities since injury correlated with higher mental HRQOL
Grauwmeijer et al., (2014)	Age Age was a predictor for physical HRQOL Functional independence Functional independence was a predictor for physical and mental HRQOL Length of hospital stay Length of hospital stay was a predictor for physical and mental HRQOL TBI severity TBI severity was a predictor for mental HRQOL Time since injury Time after injury was a predictor for physical HRQOL	Psychiatric disorders Psychiatric disorders were a predictor for mental HRQOL	Discharge destination Discharge destination was a predictor for physical HRQOL
Grauwmeijer et al., (2018)		Depression Participants with depression had worse scores in all SF-36 domains, except Physical Functioning and Bodily Pain	
Gregorio et al., (2014)		Coping styles An increase in non-productive coping styles (passive reactions/avoidance strategies) were associated with lower QOL	
Henry et al., (2006)	Difficulty in identifying emotions (alexithymia) Participants who had alexithymia reported poorer QOL		
Hibbard et al., (2004)		Depression At the 12-month follow-up, individuals with no depression had higher QOL than the chronic or late onset depression groups, and an equivalent rating with the resolved depression group	
Huebner et al., (2003)	Disability (activity restriction) Individuals who had fewer disabilities had higher QOL		Community integration Individuals who had more community integration had higher QOL
Jacobsson et al., (2010)	Sex Males reported lower scores on most of the SF-36 domains except for Role-Emotional and Mental Health domains Time since injury Participants who had a longer time since injury reported better HRQOL	Self-appraisal Self-appraisal was a predictor of physical HRQOL	Marital status Participants who were single or divorced had lower HRQOL in most domains Productive activities Participants who were not engaged in a productive activity (studying or working) had lower HRQOL in most domains. Engagement in a productive activity was a predictor of physical HRQOL
Johnson et al., (2020)	Symptom severity Severity of symptoms was negatively correlated with QOL	Self-efficacy and self-determination (mastery) Self-efficacy, and self-determination were correlated positively with QOL. Mastery was moderately associated with QOL	Social support Social support was correlated positively and moderately associated with QOL
Kalpakjian et al., (2004)		Positive affect Positive affect was associated with good QOL	Community integration Community integration was associated with good QOL Social support Social support was associated with good QOL
Koskinen et al. (1998)			Friendship Participants who reported a decrease in friendships had low QOL Intimate relationships Participants who reported a decrease in intimate relationships had low QOL Activities (leisure) Participants who reported a decrease in leisure activities had low QOL
McLean et al., (2014) Canada			Social participation Enjoyment with social participation Satisfaction with social participation Satisfaction and enjoyment with performance, and higher proportion of activities performed with others were positively correlated with QOL
Nalder et al., (2012)			Transition success from hospital to home Increased transition success was correlated with high HRQOL
O'Neill et al., (1998)			Employment Being employed was correlated with higher QOL
Pettemeridou et al., (2020)	Cognition (executive functioning) Individuals with lower executive functioning reported higher scores on the social relationship domain of QOLIBRI	Self-awareness Participants with lower self-awareness reported higher QOL on cognition, self, and the total score of the QOLIRBI, and higher scores on the psychological domain of the WHOQOL-BREF	
Rauen et al., (2020)	TBI severity HRQOL was weakly associated with initial TBI severity	Anxiety Depression 36% of participants reported low HRQOL due to depression and/or anxiety	
Rauen et al., (2021)	Age Sex More females reported poorer HRQOL. Particularly older females (54 to 76 years) reported poorer HRQOL on the cognition, emotion, and self-perception subscales of the QOLIBRI		
Reddy et al., (2017)	Cognition Motor speed and visual memory were correlated with the psychological QOL domain. Additionally, there was a positive correlation between the physical, psychological, environmental, and overall QOL with category fluency and verbal delayed memory		Education The numbers of educational years were correlated with the environmental QOL domain
Sashika et al., (2017)			Social participation Participants who had difficulties in their social participation had lower HRQOL in the role/social component than participants who achieved social participation
Sasse et al., (2014)		Coping styles Action/Distraction coping strategies were weakly and positively correlated with the Self and Social Relationships of the QOLIBRI, but there were no correlations with the SF-36. Trivialisation/Resignation coping strategies were negatively correlated with all HRQOL domains	
Soberg et al., (2013)	Disabilities More disabilities predicted worse HRQOL	Anxiety Depression Higher levels of anxiety/depression predicted worse HRQOL	Employment (pre-injury) Pre-injury employment predicted better HRQOL
Steadman-Pare et al. (2001)	Sex Being a female was associated with higher QOL		Education Education correlated with higher QOL Marital status Being married correlated with higher QOL Social participation Higher participation was correlated with higher QOL and engaging in work and leisure were associated with higher QOL Social support Social support correlated with higher QOL while more availability of emotional support was associated with higher QOL
Takada et al., (2016)			Social support Family support was associated with the Role-Social component of the SF-36
Tomberg et al., (2005)		Coping styles Participants with TBI used task-oriented coping less often than controls, however the usage of task-oriented coping styles reported a moderate correlation in the domain of Physical Functioning, and weak correlations in domains of Emotional Well-being, Energy/Fatigue, Social Functioning, and General Health Optimistic life orientation Optimistic life orientation was moderately correlated with all QOL domains	Satisfaction with social support Being satisfied with social support was moderately correlated with all QOL domains except health change
Tomberg et al., (2007)	Age Age influenced QOL	Coping styles The use of avoidance strategies correlated with lower QOL in the sociality domain	Education Education influenced QOL Satisfaction with social support Satisfaction with social support influenced QOL Work adjustment Work adjustment influenced QOL
Tsaousides et al., (2008)	TBI Severity TBI severity was associated with higher QOL		Employment Higher employment was associated with higher QOL Income Income was associated with higher QOL Work discrepancy Reduced work discrepancy was associated with lower QOL
Tsaousides et al., (2009)	Age Age at injury was positively correlated with UIN and a predictor for Life-3 TBI severity Injury severity was negatively correlated with UIN and a predictor for UIN	Self-efficacy (general and employment-related) Employment related self-efficacy and general self-efficacy correlated positively with Life-3 but negatively with UIN. Employment related self-efficacy was a predictor for Life-3, while general self-efficacy was a predictor of Life-3 and UIN	Employment Employment was correlated positively with Life-3 Income Higher income was correlated with higher QOL on both QOL measures. Income was also a predictor of life-3 and UIN
Tsaousides et al., (2011)		Suicide ideation Participants who had suicide ideation reported lower QOL	
Ulfarsson et al., (2014)		Substance Use Participants who had a history of drug and alcohol abuse pre-injury reported worse HRQOL at follow-up	Employment (pre-injury) Pre-injury unemployment predicted worse HRQOL in the Physical Functioning domain Sick leave (pre-injury) A history of sick leave predicted worse HRQOL in the Physical Functioning domain
Vickery et al. (2005)		Depression Lower levels of depression were correlated with higher QOL Positive views of self More positive views of self were correlated with higher QOL	
Wielenga-Boiten et al., (2015)	Cognition Higher cognition was associated with higher total HRQOL. Cognition was also associated with psychosocial HRQOL Functional independence Higher functional independence was associated with higher total HRQOL and was associated with physical HRQOL Length of hospital stay Length of hospital stay was associated with physical HRQOL	Anxiety Depression The absence of anxiety/depression were associated with higher total HRQOL and psychosocial HRQOL Health locus of control Having a greater belief that health status was influenced by chance, was associated with lower total HRQOL. Additionally, it was associated with psychosocial HRQOL	Discharge destination (nursing home vs home) Discharge destination was associated with psychosocial and physical HRQOL. Not being discharged to a nursing home was associated with higher total HRQOL
Williams et al., (2012)	Mobility Mobility had a moderate correlation with the coping domains of the AQOL-2 measure, and strong correlations with the independent living and social domains of AQOL-2. Mobility did not correlate with the WHOQOL-BREF measure		

## Results

**Fig. 1:**
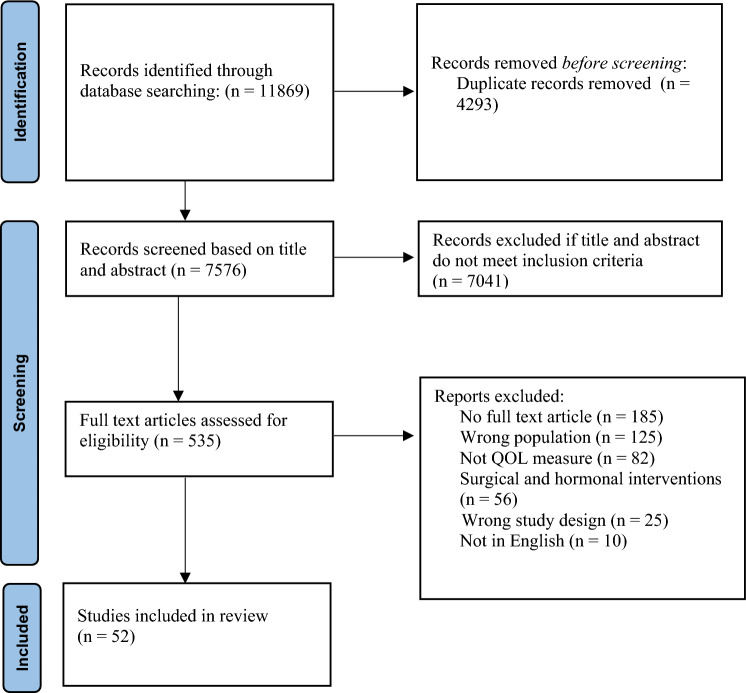
Flow diagram of study selection process

The initial database search identified 7576 articles as seen in Fig. 1 [18]. 535 articles were included in the full-text screening process, and a total of 52 articles were included in the review (Table 1). Most of the studies were from the United States of America (29%), Australia (13%), and Norway (12%). Of the included articles, 21 were cross-sectional, 20 used a prospective longitudinal design, six were case–control, four used retrospective longitudinal designs, and one followed a retrospective cross-sectional design (Table 1). The most common QOL measures were the Short Form-36 Health Survey (16 articles), Quality-of-Life After Brain Injury (6 articles), and Quality-of-Life Inventory (6 articles) (Table 1). Given the breadth of literature on QOL after TBI, our findings below are categorized based on biological, psychological, and social domains.

### Biological factors

There were 28 studies that reported 19 biological QOL factors, such as sex, TBI severity, cognition, age, time since injury, mobility, functional independence, length of hospital stay, length of ICU treatment, pre-injury comorbidities, thoracic injury, trauma, epilepsy, fatigue, sleep disturbance, symptom severity, identifying emotions, global function, and duration of posttraumatic amnesia.

There were seven studies that reported on sex and QOL after TBI [19–25]. Most studies found that females reported lower HRQOL than males [19–23]. However, two studies reported contrasting findings, where being male was associated with lower QOL [24, 25]. One study identified that males had lower scores than females on all HRQOL domains, except for the domains of role-emotional and mental health [24], while another study reported that females had higher self-rated QOL [25].

A person’s age was another biological QOL factor after TBI, with differing results [23, 26–32]. Van Delft-Schreurs et al. [30] found that higher QOL in the environmental domain was associated with older age, while Tsaousides et al., [29] identified a positive correlation between the age at injury and QOL. In contrast, one study noted that older women reported worse QOL [23], and a separate study reported that participants aged 31 and above, had decreased scores in the role-physical domain of QOL [28]. However, the relationship of age and QOL varies across the lifespan and could explain the discrepant findings looking for linear correlations or associations.

The severity of a TBI was a common QOL factor, as illustrated by six studies [21, 28, 29, 32–34]. Mixed findings were described, with two studies associating higher severity of injury with better QOL [28, 34], while one study reported high severity of injury with low QOL [29]. A separate study identified that higher severity of TBI was associated with lower scores on the physical functioning domain, but higher scores on general health and role-physical domains [21]. Additionally, one study found TBI severity to be a predictor for mental HRQOL [32], while another study found a weak correlation between TBI severity and HRQOL [33].

There were six studies that described how cognition affected QOL [27, 35–39]. Except for one study, higher scores on neuropsychological tests were associated with higher QOL. Cognition was a direct predictor of QOL [27], and people with cognitive impairments, such as deficits in attention, memory, and executive functioning reported lower QOL scores [35–37, 39]. However, a case–control study reported that those with lower executive functioning had high scores in the social relationship QOL domain [38].

There were a few factors that were identified once or by only a small number of studies. The factors reported only once included sleep disturbance, pre-injury comorbidity, longer periods of posttraumatic amnesia, epilepsy, fatigue, mobility problems, symptom severity, lower global function, thoracic injuries, and longer length of ICU treatment [19, 22, 30, 36, 40–43]. These factors impacted QOL negatively, except for thoracic injuries, which were associated with better QOL in the environmental domain. Time since injury, functional independence, identifying emotions, and longer hospital stay were identified by a few studies. Time since injury was a predictor of QOL [32, 44], while two other studies reported that as time since injury increased, so did QOL [22, 24]. There were mixed findings for identifying emotions, with one study reporting poorer QOL for those who had difficulties [45], while another study noted lower QOL for those who had better facial affect recognition [46]. Functional independence was also identified as a factor, with three studies showing associations with physical HRQOL [28, 32, 39]. Finally, longer hospitalization reported decreased QOL and was a predictor for physical functioning [30, 32, 39].

### Psychological factors

There were 31 studies that reported 16 psychological QOL factors, these included depression, anxiety, other psychiatric disorders, self-efficacy, coping styles, optimistic life orientation, positive affect, positive changes, positive views of self, self-awareness, posttraumatic stress, somatic impairments, motivation, health locus of control, suicide ideation, and self-appraisal.

Mental health disorders, such as depression, anxiety, and other psychiatric disorders, lowered QOL as identified by 18 studies. Depression was the most common mental health disorder, as depression was associated with lower QOL [27, 42, 44, 47, 48] with low scores in almost all the HRQOL subscales [19, 28, 36, 49, 50] and negative correlations with QOL [35, 51]. Additionally, a prospective longitudinal study found that individuals with no depression had higher QOL than those with late onset or chronic depression, and similar scores to those who had resolved depression [52]. Anxiety was assessed along with depression, and individuals with these disorders portrayed low QOL [33, 39, 42, 47]. Similar findings were noted for other psychiatric disorders, such as substance use, eating disorders, and personality changes [32, 47, 50, 53].

Coping styles and self-efficacy were psychological QOL factors identified in this review, which described how an individual with TBI approaches difficulties and attains goals. A case–control study reported that individuals with TBI used task-oriented coping styles less often than controls; however, individuals with this type of coping style reported higher QOL [54]. Three studies highlighted non-productive or avoidance strategies after TBI, with individuals portraying lower QOL [31, 55, 56] and one study portraying negative correlations with all domains of HRQOL [55]. Self-efficacy was reported to correlate positively with QOL by two studies [29, 41]. However, in one study, while general and employment-related self-efficacy were positively correlated with perceived QOL, there was a negative correlation with a global QOL measure [29].

Individuals who experienced optimistic life orientation, positive affect, changes, and views of self had higher QOL after TBI, while those with posttraumatic stress had lower QOL [28, 51, 54, 57–60]. Viewing life with optimism post-injury was positively correlated with all HRQOL domains [54]. Additionally, experiencing positive emotions, changes, and views of self in life post-injury also facilitated QOL [28, 51, 57, 58]. Participants with posttraumatic stress had lower QOL than individuals with no posttraumatic stress [59]. Furthermore, mental HRQOL was negatively associated with posttraumatic stress symptoms after TBI [60].

Other psychological factors that were identified in this review include self-awareness, somatic impairments, motivation, self-appraisal, and suicide ideation. With regard to self-awareness, as an individual’s awareness increased, QOL decreased [38, 48]. Somatic impairments, motivation, self-appraisal, and suicide ideation were identified once by the studies in this review. Somatic impairments and self-appraisal were predictors of QOL, while low motivation and thoughts about suicide portrayed low QOL [24, 27, 35, 61].

### Social factors

There were 31 studies consisting of 19 social factors that affected QOL, these included employment, income, work discrepancy, productive activities, work adjustment, education, community integration, social participation, social support, marital status, friendships, living with others, intimate relationships, social isolation, discharge destination, transition success, pre-injury sick leave, relatives interpersonal functioning, and environmental setting.

Employment was the most common social factor, with 10 studies stating that individuals who were employed had higher QOL [19, 21, 22, 28–30, 34, 42, 53, 62]. Individuals with pre-injury employment had higher scores in all HRQOL domains [28], and it was also a predictor for higher HRQOL [22, 42, 53]. Income, work discrepancy, engagement in productive activities, work adjustment, and education were identified as other vocational-related social factors. Income correlated and was a predictor of high QOL after TBI [29, 34], while reduced work discrepancy (e.g., how work needs are met related to perceived importance of work) was associated with low QOL [34]. Productive activities, defined as work or study programs, were another predictor of QOL [24, 63], while work adjustment correlated with QOL [31]. Individuals with more education reported better QOL [21, 25, 28, 31, 37], with some studies noting an increase in specific QOL domains such as the physical functioning [21, 28] and environmental domain [37].

Community integration and social participation increased QOL. A retrospective longitudinal study identified that more community integration and fewer activity limitations, increased QOL [64], while a cross-sectional study found that those with community integration had higher HRQOL in almost all domains [28]. Additionally, community integration was stated as a predictor for QOL [20, 57]. Social participation had a positive impact on QOL [26, 65], with one study showing that participants who socially participated in more activities with others reported high QOL [26].

Individuals who had social support, friendships, and were married had higher QOL, hence, indicating the importance of having connections after TBI. There were 7 studies that portrayed individuals with high social support reported better QOL [25, 31, 41, 54, 57, 63, 66]. Two studies noted that satisfaction of social support correlated with higher QOL [31, 54], while another study identified positive appraisals in seeking support as a predictor of high QOL [63]. A larger number of friends were a predictor for QOL [67], while losing friendships reported a low QOL score [68]. Being married and living with other individuals was highlighted by three studies in increasing QOL [24, 25, 30]. Additionally, participants who reported a low QOL were more socially isolated and had fewer intimate relationships [68].

There were six additional social factors reported, such as discharge destination, transition success, pre-injury sick leave, relatives interpersonal functioning, and environmental setting. Home discharges compared to nursing home discharges was associated with greater QOL, and increased transition success from hospital to home was correlated with higher QOL [32, 39, 69]. A history of pre-injury sick leave predicted worse QOL, relatives interpersonal functioning was associated with mental and physical HRQOL, and rural settings were a predictor for high QOL [53, 63, 70].

## Discussion

This scoping review identified 52 studies, which reported factors of QOL after sustaining a TBI. The following discussion considers these factors with regard to the biological, psychological, and social domains.

### Biological factors

Many studies identified sex as a biological factor of QOL [19–25]. However, in these studies, the sex of the participant (e.g., male, female) was reported as ‘gender.’ As we now understand gender to be a sociocultural construct [71], for this review, we have used the term sex (biological) instead. Majority of the studies indicated that females reported lower QOL [19–23], while a few identified that being male was linked to low QOL [24, 25]. Females are reported to have shorter hospital stays and receive less intensive care and rehabilitation after their TBI [72]. As such, females may be at risk for poorer long-term outcomes, and hence, experience a negative impact on their QOL. Sex was also the only biological factor that affected the same QOL domain in multiple studies. In four studies, females reported low QOL on the mental health domains of their QOL outcome measures. Previous literature shows that women with TBI are likely to experience more depressive symptoms than men or those without the injury [73–75]. Gender roles can contribute to this, as gender or power imbalances may be amplified post-injury [76, 77]. For example, if a woman occupies the role of a caregiver in their household, they may be expected to retain these duties and receive minimum assistance from others. This disparity showcases the need for support and help for women to reformulate their roles and characteristics post-injury [78].

TBI severity was a biological factor that reported mixed findings in this review. For some studies in our review, higher TBI severity was associated with higher QOL [28, 34]. Literature has noted the paradox of high severity of injury associated with better QOL, indicating that reduced awareness of injury-related deficits may be a causal factor for higher QOL [79, 80]. This also aligns with another finding from this review, as two other studies associated high self-awareness with low QOL [38, 48]. Indeed, as an individual’s self-awareness improves, the realization of deficits and consequent changes in function are more apparent, thereby decreasing their perceived QOL. There was one study that reported increasing severity resulted in lower QOL, which could be indicative of the physical and cognitive problems from a TBI [29]. However, it must be noted that the studies reporting on injury severity and QOL have most likely excluded individuals who are not able to self-report. As QOL measures are self-reported, individuals who have severe injuries from their TBI may not have the capacity to complete a QOL measure and hence, may be excluded from the study sample.

Cognitive impairment, such as deficits in attention, memory, and executive functioning, were identified as a factor that lowered QOL [27, 35–37, 39]. This review highlights the extent in which cognitive impairments impede everyday functions as various QOL domains (e.g., environmental, psychosocial) were impacted. For example, Gorgoraptis et al. [36] reported that the physical functioning, social and emotional role functioning, and mental health domains were all affected. Additionally, poor cognition is often associated with comorbidities such as sleep disorders which also lower QOL [81, 82], as highlighted in this review [36]. As such, these findings add to the literature that state the importance of improving cognition in rehabilitation and the need for effective interventions [83–85].

Fatigue, among others, were identified only once by studies in this review, indicating a gap in the research on these biological factors. Fatigue is a complex symptom with debilitating effects that is often difficult to measure objectively. Approximately up to 80% of individuals after a TBI experience fatigue [86], and it is also common in various chronic populations such as multiple sclerosis and stroke [87, 88]. While research on fatigue in the TBI population has been conducted through reviews and intervention outcomes, there is still much to explore about post-TBI fatigue and QOL.

### Psychological factors

The most common psychological factor identified was mental health disorders, with depression, anxiety, and other psychiatric disorders, with individuals reporting low QOL [19, 27, 28, 32, 33, 35, 36, 39, 42, 44, 47–53]. The risk of depression after a TBI is doubled compared to a non-TBI population [75]. The symptoms of depression after a TBI are pervasive, as in this review, most studies reported low scores in all QOL domains. This aligns with other findings in populations such as multiple sclerosis [89], cancer [90], and Parkinsons [91]. Depression is associated with poor health and social outcomes such as a decrease in social activity, occupational function, and relationship status [92, 93], and as such, these findings highlight the importance of long-term support systems and screenings that need to be available for individuals with TBI [94].

Studies in this review have identified that active or task-oriented coping strategies, such as cognitive behavior strategies, facilitates high QOL, when compared to maladaptive strategies (i.e., avoidance, trivialization) [31, 54–56]. Using coping styles that are characterized by actively working on problems have indicated positive associations with emotional adjustment and positive affect post-injury [95, 96]. Using problem-solving oriented coping styles were reported to correlate with the socially related domains in this review [54, 55]. This indicates that by developing strategies and actively working on ways to manage stressful situations, individuals may find it easier to resume their social interactions [97], and hence increase their QOL.

It was surprising that only two studies explored the effects of self-awareness on QOL [38, 48], given that up to 97% of individuals with moderate to severe TBI experience some degree of impaired self-awareness [98]. Furthermore, both studies reported varying results for different QOL domains, portraying the need for more research to enhance knowledge on the effects of self-awareness on QOL. Assessing self-awareness is an essential step in TBI rehabilitation, as those with impaired self-awareness can fail to recognize their lack of capabilities [99]. This can create challenges when trying to resume meaningful roles or participate in everyday activities, both factors which can affect QOL [25].

### Social factors

The most common social factor identified in this review was employment, with individuals who were employed having higher QOL [19, 21, 22, 28–30, 34, 42, 53, 62]. Most studies reported high scores in the physical functioning QOL domain, indicating that physical performance is a necessary factor for vocational outcomes. Notably, while returning to work is an important goal in rehabilitation [100], a longitudinal study stated that only 44% of individuals with a moderate to severe TBI return to work after 3 years post-injury due to cognitive and physical difficulties [101]. This can have a negative impact on an individual’s QOL as being employed improves many psychosocial outcomes such as self-esteem and financial independence [102, 103]. Furthermore, higher income is associated with greater QOL, as reported by this review [29, 34]. Individuals can face more expenses post-injury, such as medical costs [104]. Direct medical costs for individuals with TBI are on average $4906 higher than the medical costs for individuals with non-head injuries [105]. Earning a higher income may help these individuals manage any financial challenges and reduce finance-related stressors.

This review identified social factors of community integration, participation, and support as prominent QOL contributors. After a TBI, a ‘gap’ may be identified in activities that are more physically and cognitively challenging (e.g., return to work, playing sports) [106]. Community integration and social participation are essential after a TBI, as it facilitates the participation in new and meaningful activities, building connections, and creating new life roles [107], all of which can help improve QOL. Social support can be important in facilitating participation and also increased QOL, as highlighted in this review [25, 31, 41, 54, 57, 63, 66]. Receiving support from brain injury communities, rehabilitation programs, family, and friends provides help to navigate the new and unexpected experiences that can arise post-injury [108]. However, findings from our present review and other literature, notes that these social supports often decrease overtime, emphasizing the need for long-term supports [31, 66, 109, 110].

Transition success and discharge destination were among two social factors that were reported only a few times [32, 39, 69]. The transition post-injury from hospital to home is complex as it involves the re-integration into pre-injury settings with the added challenges that stem from a TBI (e.g., cognition, greater dependence on others). There is a need for more research about the transition phase, as increased levels of depression and stress may be experienced as individuals start to adapt to life after injury [111]. While Wielenga-Bolten et al. reported that not being discharged to a nursing home was associated with total higher HRQOL [39], individuals with moderate to severe TBI may benefit from interdisciplinary in-patient rehabilitation instead [112]. Since substantial care may be needed after a moderate to severe TBI, discharge to home may mean reduced facilities for the individual post-injury or increased caregiver burden for family members [113]. More research on understanding the meaning and lived experiences of the transition phase and being discharged to non-home settings can help identify the barriers to QOL.

### Limitations

This study had three main limitations. First, as the definition and scope of QOL are vast, there were a large volume of articles obtained in this review, and this may have impacted the rigor and specificity of our review. While we provided a broad account of the data and encompassed all literature relating to this broad construct of QOL, a detailed overview of the changes in different QOL domains have not been reported. Second, our search included only published literature, as gray literature was not explored. As such, relevant articles and theses may have been excluded and limited the depth of findings. Last, this review only included articles published in English and may have limited findings to English-speaking areas of the world. As factors relating to QOL may vary in non-English-speaking countries, excluding articles published in other languages may have limited the scope of the findings.

## Conclusion

This scoping review, consisting of 52 articles, identified studies that looked at the biopsychosocial factors of QOL after a moderate to severe TBI. Future research can explore how these biopsychosocial factors can be modulated to inform targeted rehabilitation interventions to improve QOL and further understand the subjective experiences about potential biopsychosocial factors of QOL. The data from this review will inform best practices of care and the development of novel rehabilitative interventions to improve outcomes for people after TBI.

### Supplementary Information

Below is the link to the electronic supplementary material.

Supplementary file1 (DOCX 15 KB)

## Data Availability

Not applicable.
