# Supplementary material for: Biopsychosocial factors of quality of life in individuals with moderate to severe traumatic brain injury: a scoping review
Source: Qual Life Res. 2023 Nov 5;33(4):877–901. doi: 10.1007/s11136-023-03511-0 (PMC10972932; doi:10.1007/s11136-023-03511-0)
Supplement: Supplementary file 1 — Supplementary file1 (DOCX 15 KB) [file 11136_2023_3511_MOESM1_ESM.docx]

| **Medline** | **Embase** | | **CINAHL** | | **PsycINFO** |
| --- | --- | --- | --- | --- | --- |
| **Concept 1: Traumatic Brain Injury** | | | | | |
| Terms | | | | | |
| brain injuries/ | brain injury/ | | (MH "Brain Injuries") | | DE "Traumatic Brain Injury" |
| or brain injuries, diffuse/ | or acquired brain injury/ | | or (MH "Brain Contusions") | | or DE "Brain Injuries" |
| or diffuse axonal injury/ | or brain contusion/ | |  | |  |
| or brain injuries, traumatic/ | or brain damage/ | |  | |  |
| or brain contusion/ | or traumatic brain injury/ | |  | |  |
|  | or diffuse brain injury/ | |  | |  |
|  | or diffuse axonal injury/ | |  | |  |
| Keyword and Phrases | | | | | |
| (((brain or head) adj3 (injur* or contusion)) or diffuse axonal injur*).mp. | (((brain or head) adj3 (injur* or contusion or damage)) or diffuse axonal injur*).mp. | | "brain injur*" or "head injur*" or TBI or "Brain Contusion*" or "diffuse axonal injur*" | | brain injur* or head injur* or traumatic brain injur* or acquired brain injur* |
| **Concept 2: Quality of Life** | | | | | |
| Terms | | | | | |
| "Quality of Life"/ | | "Quality of Life"/ | (MH "Quality of Life+") | DE "Quality of Life" | |
|  | |  |  | or DE "Health Related Quality of Life” | |
| Keyword and Phrases | | | | | |
| (“Quality of life” or life quality or QOL).mp. | | (“Quality of life” or life quality or QOL).mp. | "Quality of life" or “life quality” or QOL | "Quality of life" or “life quality” or QOL | |
